# Season Affects Yield and Metabolic Profiles of Rice (*Oryza sativa*) under High Night Temperature Stress in the Field

**DOI:** 10.3390/ijms21093187

**Published:** 2020-04-30

**Authors:** Stephanie Schaarschmidt, Lovely Mae F. Lawas, Ulrike Glaubitz, Xia Li, Alexander Erban, Joachim Kopka, S. V. Krishna Jagadish, Dirk K. Hincha, Ellen Zuther

**Affiliations:** 1 Max-Planck-Institute of Molecular Plant Physiology, 14476 Potsdam, Germany; schaarschmidt@mpimp-golm.mpg.de (S.S.); lfl0008@auburn.edu (L.M.F.L.); glaubitz@mpimp-golm.mpg.de (U.G.); rainbowleelx@hotmail.com (X.L.); Erban@mpimp-golm.mpg.de (A.E.); Kopka@mpimp-golm.mpg.de (J.K.); Hincha@mpimp-golm.mpg.de (D.K.H.); 2 International Rice Research Institute, Metro Manila 1301, Philippines; kjagadish@ksu.edu; 3 Institute of Crop Science, Chinese Academy of Agricultural Science, Beijing 100081, China; 4 Department of Agronomy, Kansas State University, Manhattan, KS 66506, USA

**Keywords:** high night temperature, rice, grain yield, wet season, dry season, metabolomics

## Abstract

Rice (*Oryza sativa*) is the main food source for more than 3.5 billion people in the world. Global climate change is having a strong negative effect on rice production. One of the climatic factors impacting rice yield is asymmetric warming, i.e., the stronger increase in nighttime as compared to daytime temperatures. Little is known of the metabolic responses of rice to high night temperature (HNT) in the field. Eight rice cultivars with contrasting HNT sensitivity were grown in the field during the wet (WS) and dry season (DS) in the Philippines. Plant height, 1000-grain weight and harvest index were influenced by HNT in both seasons, while total grain yield was only consistently reduced in the WS. Metabolite composition was analysed by gas chromatography-mass spectrometry (GC-MS). HNT effects were more pronounced in panicles than in flag leaves. A decreased abundance of sugar phosphates and sucrose, and a higher abundance of monosaccharides in panicles indicated impaired glycolysis and higher respiration-driven carbon losses in response to HNT in the WS. Higher amounts of alanine and cyano-alanine in panicles grown in the DS compared to in those grown in the WS point to an improved N-assimilation and more effective detoxification of cyanide, contributing to the smaller impact of HNT on grain yield in the DS.

## 1. Introduction

Rice is a staple food for more than half of the world’s population and the demand is steadily increasing with the growing human population [1]. Climate change is a significant limiting factor for enhancing food production, because increasing abiotic and biotic stresses negatively affect the yield of all major crops [2,3,4]. During the past century, the global surface temperature has increased by an average of 0.85 °C, and a further increase of up to 3.7 °C has been predicted by 2100 [3]. This temperature increase develops asymmetrically, with a faster rise in daily minimum compared to daily maximum temperatures [5,6,7,8,9], leading to “high night temperature” (HNT) conditions. Asymmetric warming causes a reduction in the temperature difference between daily maximum and minimum temperatures, i.e., the diurnal temperature range (DTR), with a negative influence on both wild and crop plant species [10]. In particular, the main rice-growing countries in Asia, including China [11], the Philippines [12,13] and India [14,15], are affected.

Several studies have reported a strong decrease in yield and grain quality, such as increased chalk formation, and altered grain growth dynamics in rice under HNT [16,17,18,19,20,21]. HNT can have a stronger impact on grain weight than high day temperatures in rice and wheat [22,23,24]. Field studies at the International Rice Research Institute (IRRI) in the Philippines showed that rice grain yield was reduced by 10% per 1 °C increase in night temperatures during the dry season (DS), whereas the effect of increasing day temperatures was not significant within the investigated time period [12].

Differences in HNT sensitivity among various rice cultivars based on grain yield [25,26,27], yield-related parameters, or phenotypes in the vegetative stage [28] have been reported, indicating natural variation in HNT tolerance. In addition, HNT reduces the starch content in panicles and negatively affects grain yield and quality (chalk and amylose content) in the sensitive cultivars Gharib and IR64, but not in the tolerant cultivar N22 [29].

Different factors may cause HNT sensitivity. Physiological effects reported under HNT include higher rates of respiration in leaves [28,30,31] and panicles [29], whereas photosynthesis is not affected [28] or may be decreased as well [32]. A reduction in nitrogen and carbohydrate translocation after flowering as a possible cause of yield reduction in HNT sensitive cultivars was also discussed [25]. Reduced grain weight and quality may be caused by lower sink strength due to lower cell wall invertase and sucrose synthase activity in sensitive cultivars, accompanied by higher sugar accumulation in the rachis [29].

Despite the increasing knowledge of the physiological responses to HNT, only little is known about the metabolomic responses of rice under these conditions. The metabolic status is important for growth, development and stress tolerance, and additionally influences important traits such as flavor, biomass, yield and nutritional quality [33,34,35]. Therefore, the assessment of the metabolomic status of wild and crop species can help to evaluate natural variation [33]. Additionally, the metabolome integrates molecular and environmental effects as endpoints of biological processes [36]. Moreover, metabolites constitute potential markers for the selection of tolerant crop genotypes in breeding programs. Several studies investigated metabolic changes in rice in response to abiotic stress conditions, such as salinity [37,38,39,40,41], osmotic stress [42], drought [43,44,45,46,47], heat [44,48], and combined drought and heat stress conditions [49,50].

In a corresponding study on rice under HNT conditions, sucrose and pyruvate/oxaloacetate-derived amino acids were shown to accumulate while sugar phosphates and organic acids involved in glycolysis/gluconeogenesis and the tricarboxylic acid (TCA) cycle decreased in developing caryopses [48]. A dysregulation of central metabolism and an increase in polyamine biosynthesis was described for sensitive cultivars, whereas existing metabolic pre-adaptation under control conditions was found for tolerant cultivars [51,52]. Furthermore, in sensitive cultivars, 4-amino butanoic acid (GABA) signaling—and in tolerant cultivars, the jasmonate precursor *myo*-inositol—were linked to the HNT responses [52]. A metabolomics study investigating early seed development and the early grain-filling stage in six rice cultivars reported a sugar accumulation peak seven days after flowering and 19 significantly different metabolites under HNT compared to under control conditions, with a special focus on the generally higher abundance of sugars and sugar alcohols under HNT [53].

The goal of this study was to investigate the seasonal effects of HNT responses by assessing the metabolic responses to HNT stress in flag leaves and panicles during the DS and wet season (WS) in contrasting rice cultivars under field conditions. Previous studies of the comparison of HNT’s effects during the WS and DS were limited to agronomic traits [13,14,20,26,54], while the influence of HNT on the rice metabolome has not been reported yet. The present study sheds new light on the responses of rice to an important climatic stress factor that may severely limit grain yield and quality, and therefore the global food supply.

## 2. Results

Two field experiments were performed at the IRRI in the Philippines during the WS and DS with eight rice cultivars (Table 1). These cultivars comprised the *indica* and *japonica* subspecies and included HNT tolerant, intermediate and sensitive cultivars, as determined during the vegetative growth stage from a study under controlled environmental conditions [28].

**Figure A1:**
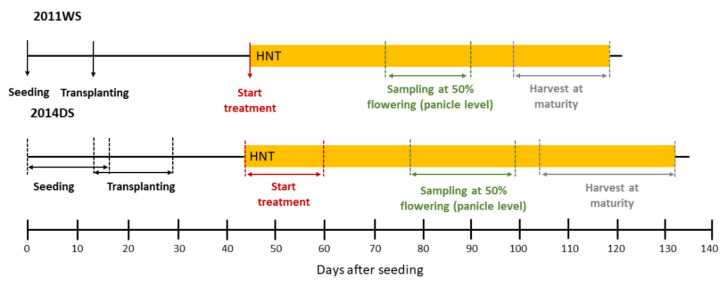
Experimental set-up for the DS and WS experiment. WS—wet season, DS—dry season.

**Figure A2:**
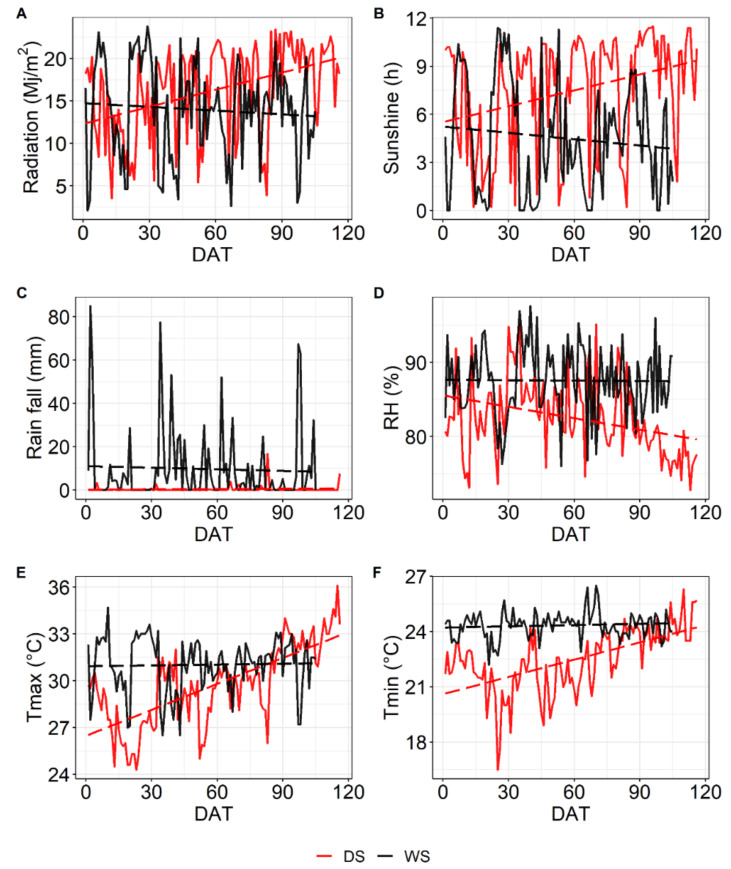
Weather data for the DS and WS experiment measured at the IRRI weather station as average values per day. Radiation (**A**), sunshine duration (**B**), rainfall (**C**), relative humidity (**D**), maximal temperature T_max_ (**E**), minimal temperature T_min_ (**F**). Broken lines represent a trend line for the respective data set. DAT—days after transplanting. Average values for all weather parameters were significantly different (*p* < 0.05) between WS and DS.

The WS experiment was performed for 84 to 104 days from transplanting till maturity, and the DS experiment, for 87 to 118 days, depending on the staggered sowing (Figure A1). Samples for metabolite analysis were taken at 59 to 78 days after transplanting in the WS, and in the DS, between 58 and 88 days. During the day (6 a.m.–6 p.m.), plants were exposed to ambient conditions, with an average temperature of 27.7 °C during the WS and 26.1 °C during the DS. The mean daytime temperature ranged from 25.4 to 29.7 °C during the WS, and from 21.8 to 30.9 °C during the DS, with maximum daily temperatures from 26.5 °C to 34.7 °C during the WS and from 24.3 °C to 36.1 °C during the DS (Figure A2e).

**Figure 1:**
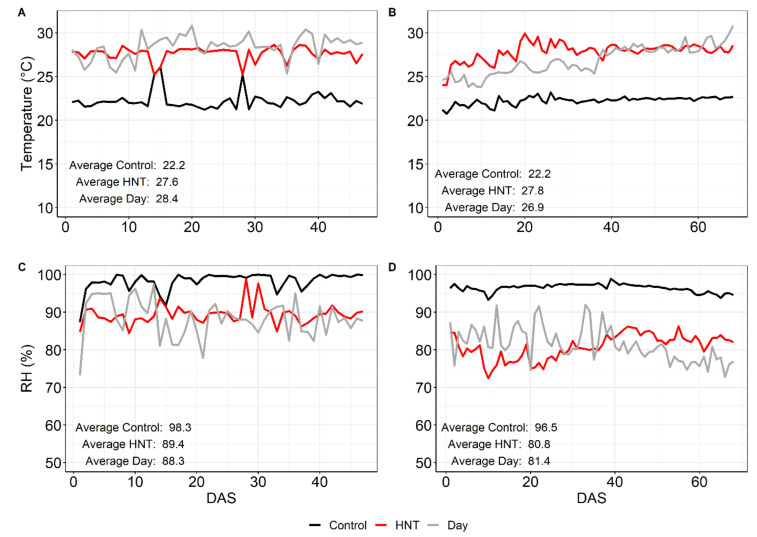
Average temperature (**A**,**B**) and relative humidity (RH) (**C**,**D**) during the night (6 p.m.–6 a.m.) in the wet season (WS) (**A**,**C**) and dry season (DS) (**B**,**D**) under control and HNT conditions, measured till the end of sampling at 50% flowering. For comparison, day temperature and humidity are included (grey lines). Measurements, which were done every 30 min, were averaged. DAS—Days after stress; WS—wet season; DS—dry season.

During the night, plots were covered by tents, and the temperature was kept constant by air conditioners, set to 22 °C for the control and 28 °C for HNT conditions. The average temperatures measured in the tents were 27.64 °C (± 0.77 °C) and 27.82 °C (± 1.07 °C) under HNT conditions and 22.24 °C (± 0.99 °C) and 22.25 °C (± 0.46 °C) under control conditions during the WS and DS, respectively (Figure 1A,B). The corresponding ambient night temperatures outside the tents are shown in Figure A2f. As the average day temperatures for both seasons were very similar, the night temperature difference of around 5 °C is the main temperature factor driving the physiological and metabolic changes in all cultivars.

Average radiation was about 22% lower in the WS than in the DS and sunshine duration in the WS reached only 45% of the values measured in the DS (Figure A2A,B). Daily rainfall in the WS was recorded between 85 and 0 mm, while it was approximately zero in the DS (Figure A2C. Accordingly, average relative air humidity was lower in the DS with values between 73% and 95%, compared to those between 76% and 98% in the WS (Figure A2D).

### 2.1. Influence of HNT on Agronomic Parameters

**Figure 2:**
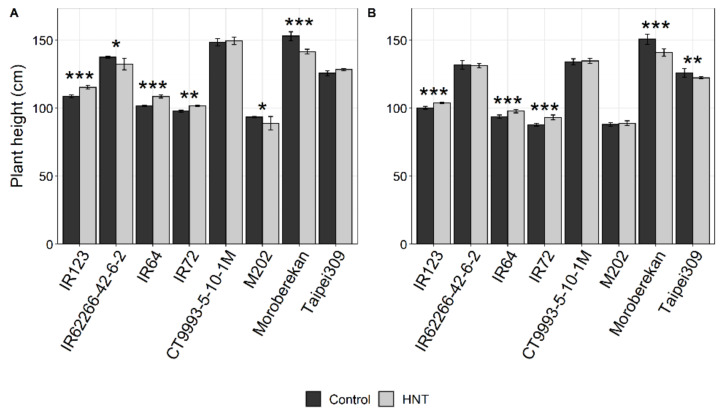
Plant height of the investigated rice cultivars under control and HNT conditions in the WS (**A**) and DS (**B**). Bars for the WS represent means ± SEM of 24 plants per condition, and bars for the DS, those of 60 plants per condition. Cultivars are sorted alphabetically within the respective *O. sativa* subspecies *indica* (1–4) and *japonica* (5–8). Significance levels are indicated by asterisks: 0.001 < ***; 0.001 > ** < 0.01; 0.01 > * < 0.05.

For all agronomic parameters, a significant genotype effect was found in both seasons when comparing samples from plants grown under HNT with control conditions (Table 2). Furthermore, a significant seasonal effect was recorded for almost all agronomic parameters. The influence of HNT conditions on the growth response was recorded as differences in plant height. No significant treatment effect but a significant Genotype x Treatment (GxT) effect of HNT on plant height was found over all cultivars for both seasons (Table 2). On average, plant height was slightly lower in the DS compared to in the WS, but cultivar-specific patterns were conserved (Figure 2). In both seasons, plant height was significantly (*p* < 0.05) increased under HNT in three cultivars (IR123, IR64 and IR72), while it was decreased in Moroberekan. IR62266-42-6-2 and M202 showed reduced plant height only in the WS, and Taipei309, only in the DS.

**Figure 3:**
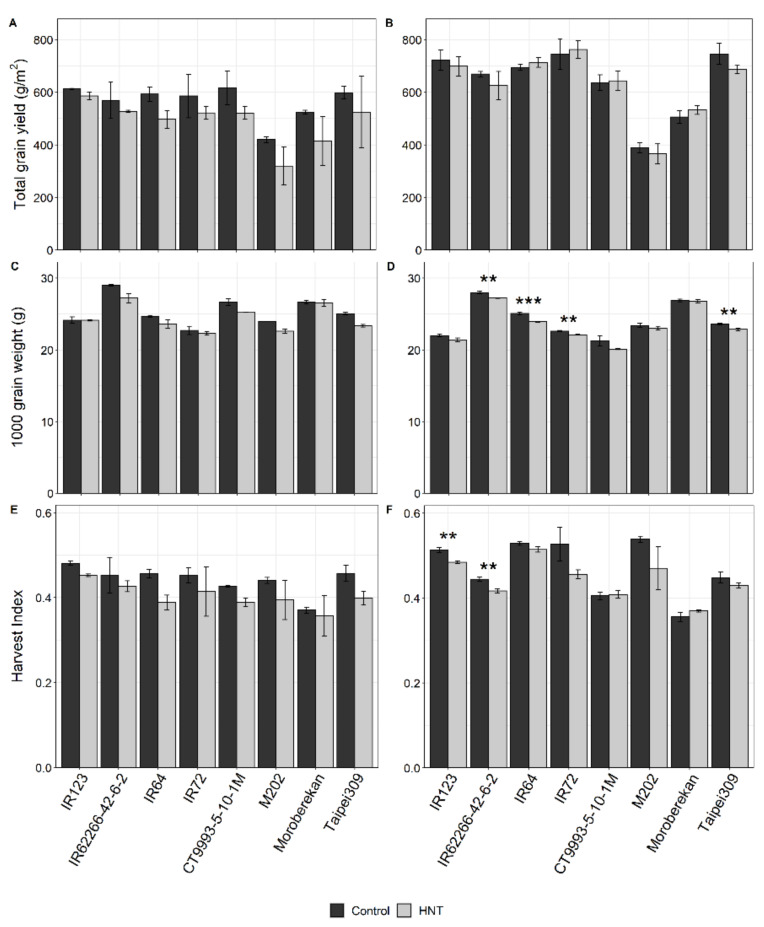
Grain yield (**A**,**B**), 1000-grain weight (**C**,**D**) and harvest Index (**E**,**F**) of eight rice cultivars under control and HNT conditions in the WS (**A**,**C**,**E**) and DS (**B**,**D**,**F**). For the WS, bars represent the means and error bars, the range of two replicates generated from 12 plants, each. For the DS, the bars represent the means ± SEM of five replicates generated from 12 plants, each. Cultivars are sorted alphabetically within the respective *O. sativa* subspecies *indica* (1–4) and *japonica* (5–8). Significance levels were only calculated for the DS due to an insufficient replicate number in the WS and are indicated by asterisks: 0.001 < ***; 0.001 > ** < 0.01; 0.01 > * < 0.05.

**Figure A3:**
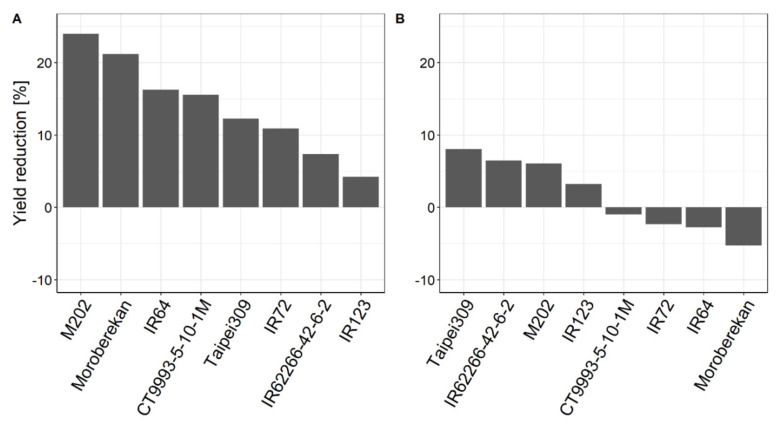
Yield reduction under HNT in the WS (**A**) and DS (**B**). Cultivars are sorted from highest to lowest yield reduction.

Total grain yield under control conditions was significantly lower in the WS, with a maximum yield among all cultivars of about 617 g·m^−2^ compared to that in the DS of 762 g·m^−2^ (Figure 3A,B). A significant effect of HNT treatment on the grain yield of all eight cultivars compared to control was only detectable in the WS (Table 2), where yield reduction varied between 23% in M202 and 4% in IR123 (Figure A3A). In the DS, yield was only reduced between 8% and 3% in four cultivars, while it was slightly increased (1%–5%) in the other four (Figure A3B). No correlation was found between the yield reduction in our experiments in the WS or DS and the HNT sensitivity rank of the same cultivars in the vegetative stage under controlled environmental conditions determined for the same cultivars in a previous study [28] (not shown).

A significant negative HNT treatment effect was also found for the 1000-grain weight in both growth seasons (Table 2), with the highest reductions in the WS of about 1.7 and 1.8 g for Taipei309 and IR62266-42-6-2, respectively (Figure 3C,D, Table 2).

**Figure A4:**
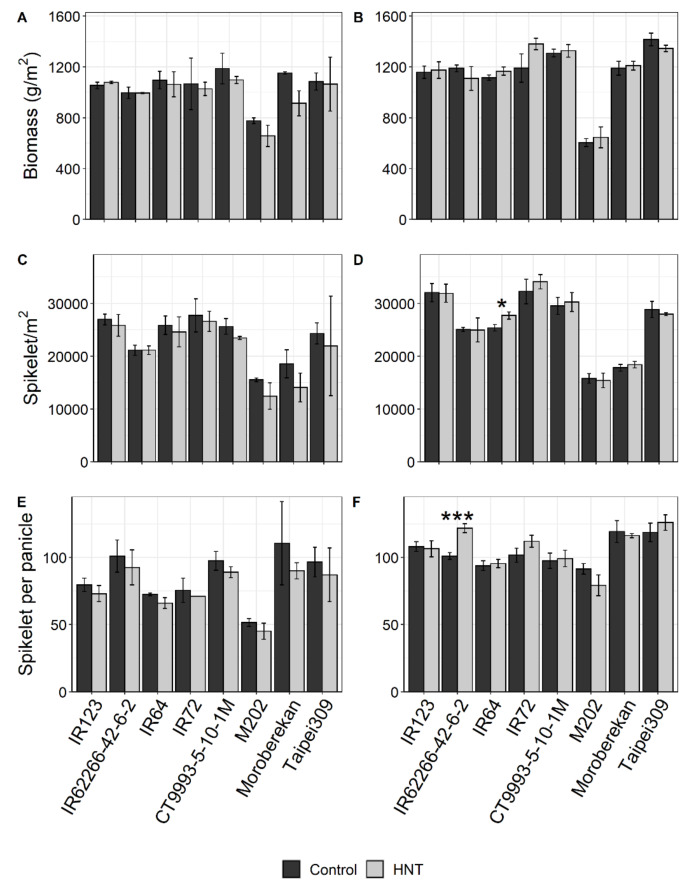
Biomass (**A**, **B**), spikelets per m^2^ (**C**, **D**), and spikelets per panicle (**E**, **F**) of eight rice cultivars in response to HNT stress for the WS (**A**, **C**, **E**) and DS (**B**, **D**, **F**). For the WS, variance is displayed as range between means of two replicates with 12 plants each; for the DS, the standard error of the mean of five replicates with 12 plants each is shown. Cultivars are sorted alphabetically within the respective *O. sativa* subspecies *indica* (1-4) and *japonica* (5–8). Significance levels were only calculated for the DS due to the insufficient replicate number in the WS and are indicated by asterisks: 0.001 < ***; 0.001 > ** < 0.01; 0.01 > * < 0.05.

The harvest index was significantly affected by HNT across all cultivars in both seasons (Table 2) and showed an overall reduction, except for Moroberekan in the WS and DS and CT9993-5-10-1M only in the DS (Figure 3E,F, Table 2). Furthermore, a significant treatment effect was determined for biomass, spikelets per m^2^ and spikelets per panicle only in the WS, but not in the DS (Table 2). For cultivar-specific changes in these parameters, see Figure A4.

### 2.2. HNT’s Effects on the Metabolome Are More Pronounced in Panicles Than in Flag Leaves

**Figure 4:**
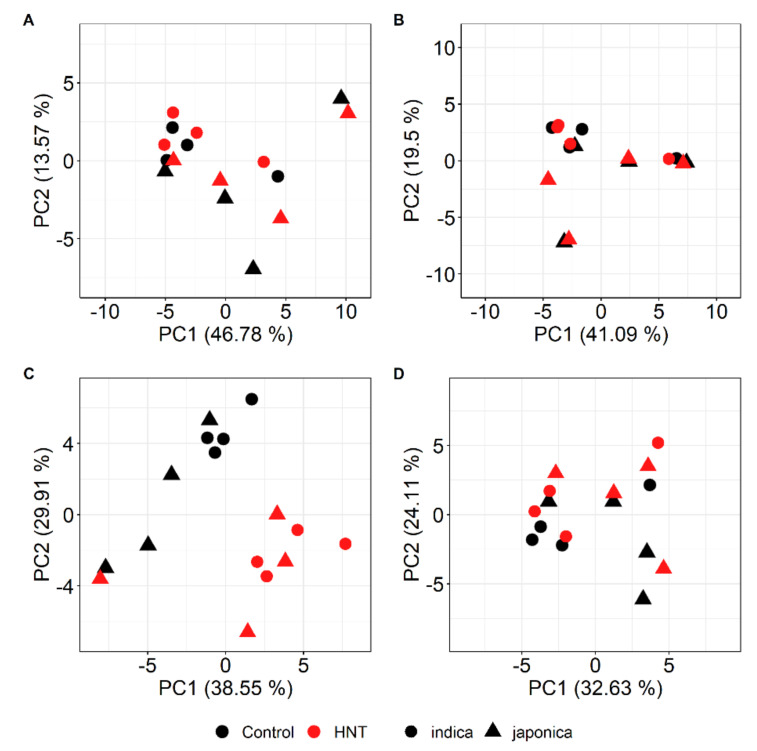
Score plots of the first two Principal Components (PC1 and PC2) from the Principal Component Analysis (PCA) of the metabolite profiles of rice flag leaves (**A**, **B**) and panicles (**C**, **D**) of the eight investigated rice cultivars under control and HNT conditions in the WS (**A**, **C**) and DS (**B**, **D**). For flag leaves, means of the median-normalized and log_2_-transformed mass spectral intensities of 76 metabolites, and for panicles, those of 69 metabolites, were used. Numbers in parentheses indicate the fractions of the total variance explained by the respective PCs.

Profiling of hydrophilic small metabolites was performed by gas chromatography-mass spectrometry (GC-MS) on flag leaves and panicles of all eight cultivars grown in both seasons. Since it has been shown previously that the metabolite profiles of rice flag leaves and panicles differ widely, making meaningful direct comparisons impossible [49], we treated the data from the two organs separately. After the pre-processing of both data sets, a total of 76 metabolites for flag leaves and 69 for panicles were determined that were detected in both seasons. Principal Component Analysis (PCA) indicated that metabolite profiles of flag leaves were not strongly affected by HNT conditions in either the WS or DS (Figure 4A,B).

Instead, a separation between cultivars belonging to the subspecies *indica* and *japonica* was visible for both seasons and treatments. By contrast, a clear separation along PC1, explaining 38.55% of the total variance in the data set, between samples from plants grown under control or HNT conditions was observed for panicles collected in the WS (Figure 4C). The single outlier represents the *japonica* cultivar Moroberekan under HNT conditions. For the DS experiment, samples from panicles under different night temperature conditions were separated by PC2, explaining 24.11% of the variance, while PC1 separated the subspecies, explaining 32.63% of the total variance (Figure 4D).

**Figure 5:**
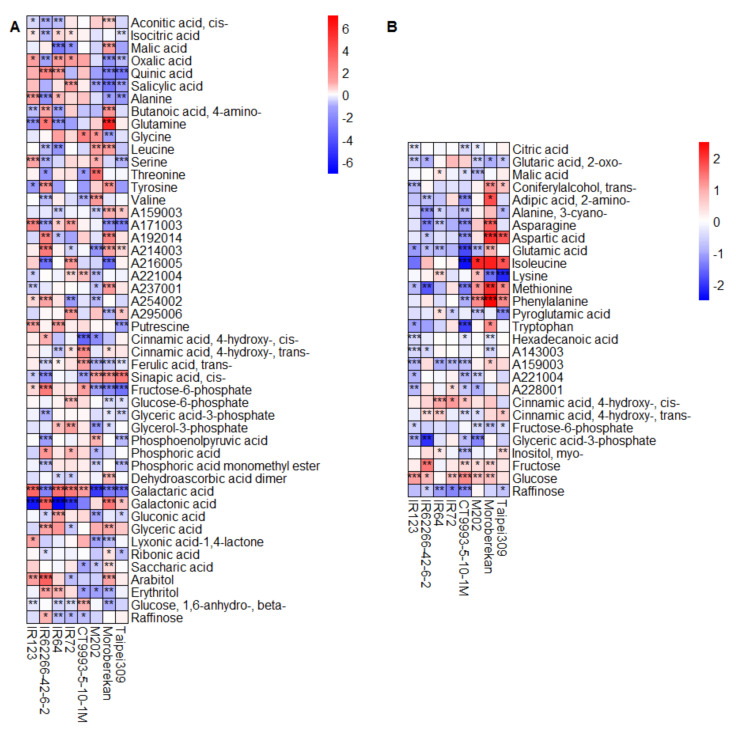
Heat maps showing the log_2_ fold changes in metabolite levels under control conditions in the DS compared to the WS for flag leaves (**A**) and panicles (**B**). Only metabolites with a significant change in at least three out of the eight cultivars are displayed. The level of significance is indicated by asterisks (* *p* < 0.05; ** *p* < 0.01; *** *p* < 0.001) and the log_2_ fold change is represented by the indicated color code. Blue indicates a lower metabolite level in the DS compared to the WS, and red, a higher level. Metabolites are listed alphabetically within the metabolite classes (compare Supplementary Table S2). Cultivars are sorted alphabetically within the respective *O. sativa* subspecies *indica* (1–4) and *japonica* (5–8).

The metabolite composition already varied under control conditions between the two growth seasons in both flag leaves and panicles (Figure 5). Of the 76 metabolites identified in flag leaves, 48 (63%) showed a significantly different content in at least three cultivars in this analysis, while of the 69 metabolites in panicles, 28 (41%) differed between seasons. Only eight of these metabolites (malic acid, A159003, A221004, cis-4-hydroxycinnamic acid, trans-4-hydroxycinnamic acid, fructose-6-phosphate, glyceric acid-3-phosphate and raffinose) were identical in both organs, indicating highly organ-specific metabolic reactions to seasonal variations in rice. In addition, there was variation among the cultivars, which was, however, largely independent of the subspecies that the cultivars belong to.

**Figure 6:**
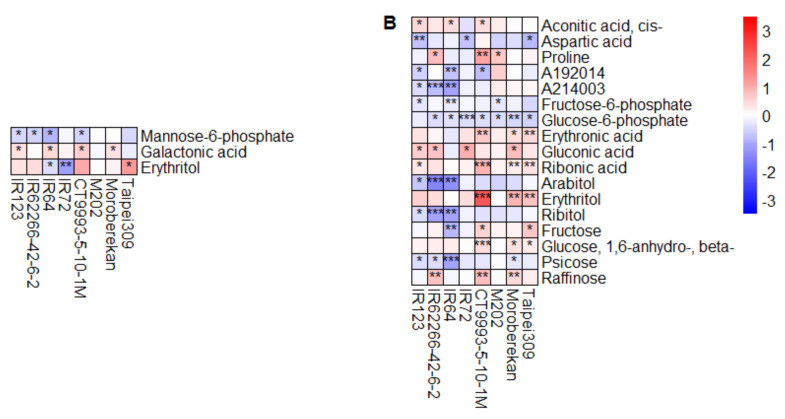
Heat maps showing the log_2_ fold changes in metabolite pool sizes in flag leaves under HNT compared to control conditions for the WS (**A**) and DS (**B**). Only metabolites with a significant change in at least three out of the eight cultivars are displayed. The level of significance is indicated by asterisks (* *p* < 0.05; ** *p* < 0.01; *** *p* < 0.001), and the log_2_ fold change is represented by the indicated color code. Blue indicates a lower metabolite level under HNT compared to under control conditions, and red, a higher level. Cultivars were sorted alphabetically within the respective *O. sativa* subspecies *indica* (1–4) and *japonica* (5–8).

Under HNT conditions, only three metabolites in flag leaves were significantly changed relative to control values in at least three cultivars in the WS, compared to 17 metabolites that were so in the DS (Figure 6). Only erythritol was significantly affected by HNT in both growth seasons. However, while it was increased or unchanged in the DS, it showed a cultivar-specific increase (strongest in Taipei309) or decrease (strongest in IR72) in the WS. In the DS, all metabolites were either reduced/unchanged or increased/unchanged across all cultivars, except for fructose, which was significantly increased in Taipei309 and CT9993-5-10-1M, and significantly decreased in IR64. In addition, while most metabolites showed significant changes in only three or four cultivars, glucose-6-phosphate was significantly reduced under HNT conditions in seven out of the eight cultivars (Figure 6B).

**Figure 7:**
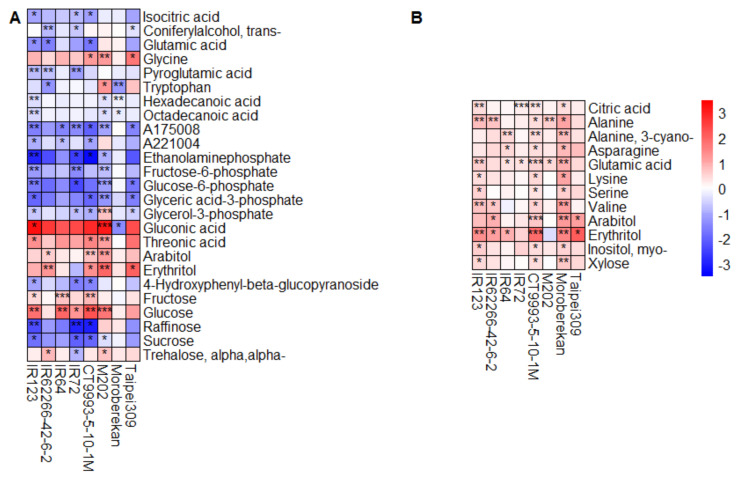
Heat maps showing the log_2_ fold changes in metabolite pool sizes in panicles under HNT compared to control conditions for the WS (**A**) and DS (**B**). Only metabolites with a significant change in at least three out of the eight cultivars are displayed. The level of significance is indicated by asterisks (* *p* < 0.05; ** *p* < 0.01; *** *p* < 0.001), and the log_2_ fold change is represented by the indicated color code. Blue indicates a lower metabolite level under HNT compared to control conditions, and red, a higher level. Cultivars were sorted alphabetically within the respective *O. sativa* subspecies *indica* (1–4) and *japonica* (5–8).

In panicles, metabolite changes caused by HNT conditions were more pronounced than in leaves, with higher log_2_ fold changes and a larger number of significantly changed metabolites—25 during the WS and 12 during the DS. In addition to the larger number of metabolites that were significantly affected by HNT in the WS than in the DS, opposite to what we observed in flag leaves (Figure 6), changes were generally also larger in the WS than in the DS in panicles (Figure 7).

**Figure A5:**
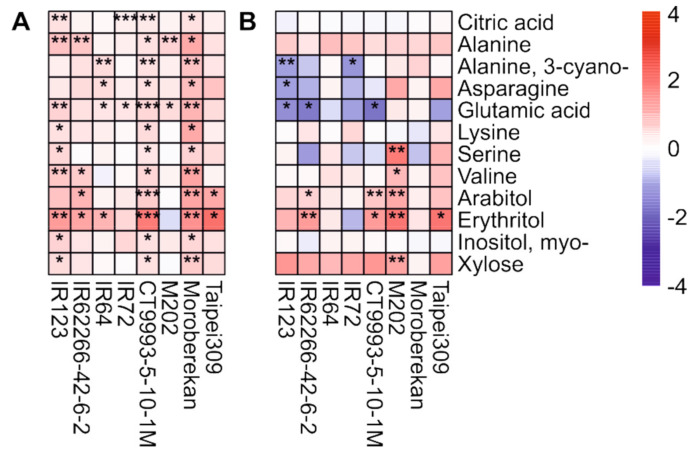
Log_2_ fold changes in significantly changed metabolite pools under HNT compared to under control conditions in panicles for the DS (**A**). For comparison, the same metabolites are shown for the WS (**B**) independent of a significant change. For the DS, only metabolites that showed a significant change in at least three out of eight cultivars are displayed in (**A**). The level of significance is indicated by asterisks (* *p* < 0.05; ** *p* < 0.01; *** *p* < 0.001), and the log_2_ fold difference is indicated by the color code. Blue indicates a lower metabolite level compared to under the control condition, and red, a higher level. Cultivars were sorted alphabetically within the respective *O. sativa* subspecies *indica* (1–4) and *japonica* (5–8).

A comparison of the significantly changed metabolites in at least three of the eight cultivars in the DS with those in the WS revealed an overlap of glutamic acid, arabitol and erythritol (Figure 7, Figure A5). Glutamic acid content was mainly reduced in the WS but increased in the DS, while the polyols arabitol and erythritol were mainly increased by HNT in both seasons. There was very little overlap in the metabolites significantly affected by HNT between flag leaves and panicles, with only erythritol affected in the WS and arabitol and erythritol, in the DS. Interestingly, arabitol showed an opposite behavior in response to HNT in the two organs, with decreased levels in flag leaves and increased levels in panicles.

**Figure A6:**
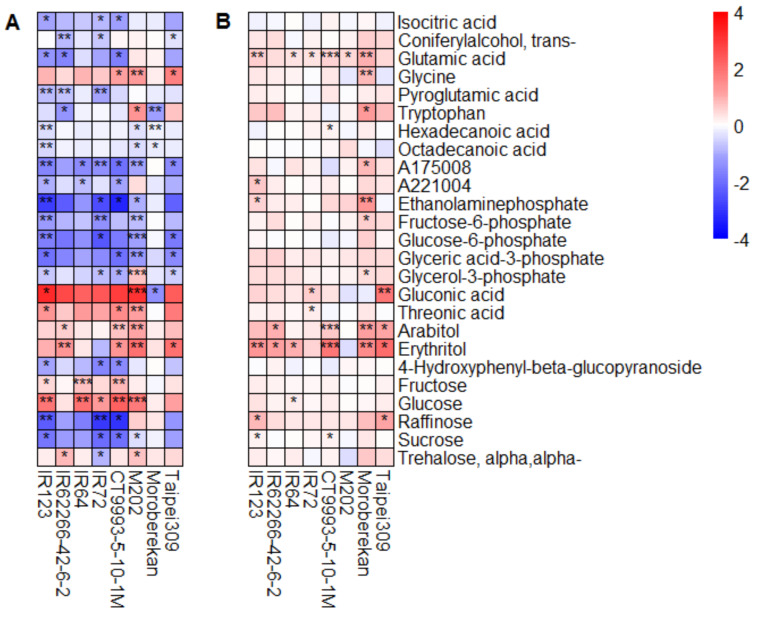
Log_2_ fold changes in significantly changed metabolite pools under HNT compared to under control conditions in panicles for the WS (**A**). For comparison, the same metabolites are shown for the DS (**B**) independent of a significant change. For the WS (**A**), only metabolites that showed a significant change in at least three out of eight cultivars are displayed. The level of significance is indicated by asterisks (* *p* < 0.05; ** *p* < 0.01; *** *p* < 0.001), and the log_2_ fold difference is indicated by the color code. Blue indicates a lower metabolite level compared to under the control condition, and red, a higher level. Cultivars were sorted alphabetically within the respective *O. sativa* subspecies *indica* (1–4) and *japonica* (5–8).

In the WS, the levels of organic acids; amino acids (except glycine); the phosphorylated intermediates fructose-6 phosphate, glucose-6-phosphate, glyceric acid-3-phosphate and glycerol-3-phosphate; and the sugars raffinose and sucrose were in general reduced during HNT in panicles compared to under control conditions. On the other hand, glycine, gluconic acid, threonic acid, arabitol, erythritol, and fructose and glucose were increased (Figure 7A). In a direct comparison of these significantly changed metabolites in the WS with the metabolite levels in the DS, no reduction of any of these metabolites could be observed in the DS (Figure A6). In the DS, all 12 of the significantly influenced metabolites (mainly amino acids, arabitol, erythritol, citric acid, glutamic acid and xylose) were increased under HNT conditions (Figure 7B).

**Figure 8:**
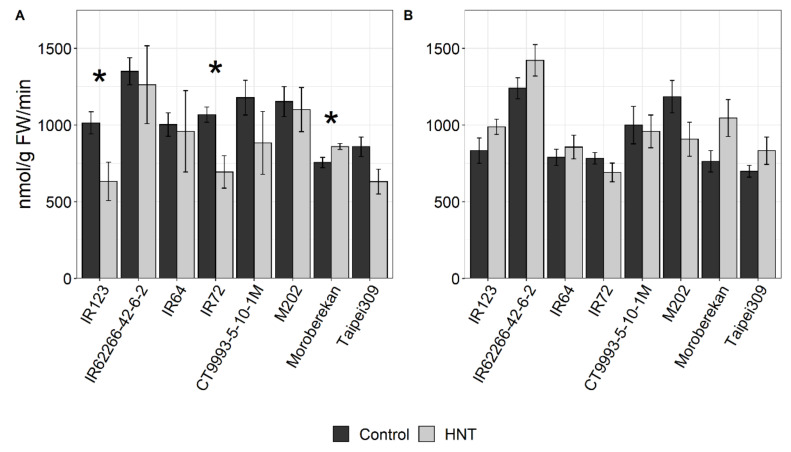
Activity of the enzyme alanine aminotransferase (AlaAT) in panicles of the indicated rice cultivars under control and HNT conditions for the WS (**A**) and DS (**B**). Values are averages of three replicates per cultivar and condition, with four exceptions with two replicates. The level of significance is indicated by asterisks (* *p* < 0.05; ** *p* < 0.01; *** *p* < 0.001). Cultivars were sorted alphabetically within the respective *O. sativa* subspecies *indica* (1–4) and *japonica* (5–8).

Alanine and 3-cyano alanine were among the metabolites that were significantly changed under HNT conditions in panicles in the DS, but not in the WS. Alanine is a major storage amino acid under stress conditions [55], and the activity of the alanine biosynthetic enzyme alanine aminotransferase (AlaAT) can influence rice yield [56]. In the WS, the activity of AlaAT was generally reduced under HNT to values of 62% to 96% (except for Moroberekan) compared to under control conditions, which was significant at *p* < 0.05 for IR123 and IR72 (Figure 8A). By contrast, AlaAT activity in the DS reached values of 77% to 137% higher under HNT in comparison to under control conditions and was increased in five out of the eight cultivars, although none of the differences were statistically significant (Figure 8B).

**Figure 9:**
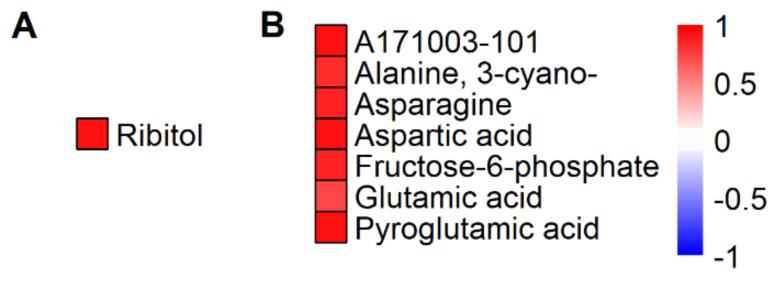
Metabolites with significant correlations (Spearman’s rank correlation, *p* < 0.05) between total grain yield reduction under HNT in the WS and the corresponding changes in metabolite contents (log_2_ fold change) in flag leaves (**A**) and in panicles (**B**). Red color indicates positive correlations. Metabolites are sorted alphabetically.

To obtain insight into the potential function of particular metabolites in HNT tolerance in the field and to identify possible candidate marker metabolites for HNT tolerance, we performed correlation analyses between the grain yield reduction in eight cultivars under HNT compared to under control conditions and the change in relative metabolite pool sizes (log_2_ fold change) under HNT in the WS, wherein HNT significantly affected grain yield. While we only identified one significant correlation for metabolites detected in flag leaves (ribitol), we found seven such correlations among panicle metabolites (Figure 9). In addition to one yet unidentified compound, the others comprised four amino acids (including 3-cyano alanine), pyroglutamic acid (representing the sum of pyroglutamate, glutamine and glutamate pools) and fructose-6-phosphate. All eight metabolites showed positive correlations, i.e., a larger change in metabolite pool size indicates a smaller yield loss.

## 3. Discussion

The response of agronomic parameters and metabolic patterns to HNT have been analyzed for eight rice cultivars with different HNT tolerance under field conditions at the IRRI in two different seasons. A comparison of the weather data for both seasons and the respective agronomic parameters identified a slightly longer time to maturity in the DS than in the WS as an important difference. During the DS, plants were exposed to higher radiation intensity and sunshine duration, but lower rainfall and relative humidity compared to in the WS. Similar differences for radiation and sunshine have been reported for a comparison of the DS and the WS from 2005 to 2009 at the IRRI [13]. Furthermore, temperature data for the two growth seasons largely agree between our study and two earlier reports for the same location [13,20], indicating that the plants in our study were exposed to normal climatic conditions without any extreme weather events.

Under control conditions, total grain yield was higher for most cultivars in the DS than in the WS, in agreement with published data [13]. Under HNT conditions, no clear changes in grain yield were observed during the DS, while it was reduced to different degrees in all cultivars in the WS. Under controlled environmental conditions, a yield reduction caused by HNT was previously observed for the cultivars IR62266-42-6-2 and CT9993-5-10-1M, while IR123 showed no change, and IR72 even showed an increased grain yield [28]. During the WS, under our field conditions, IR62266-42-6-2 and CT9993-5-10-1M also showed clear yield reductions of about 22% and 12%, respectively. However, IR123 and IR72 behaved differently under field than under climate chamber conditions, with yield reductions of 16% and 11%, respectively, emphasizing the need for field experiments to determine the effects of stress treatments on rice yield.

A similar influence of the growing season on yield reduction under HNT was previously reported for the *indica* cultivar Gharib and six tropical hybrid cultivars [20]. Additionally, for the tolerant *aus* cultivar N22, a significantly lower yield under HNT was only recorded in the WS. This yield variation was mainly attributed to a reduced grain weight and number of spikelets per m^2^, parameters also with significant negative treatment effects during the WS in the present study. Grain yield was reduced in both seasons by around 11% under HNT except for tolerant cultivars in four consecutive years, again partially attributable to a decrease in grain weight [26]. Other authors also highlighted the combination of decreased grain weight, spikelet number per panicle, and biomass production together with a decreased seed set as important for the decline in grain yield under HNT [57]. In general, a reduction in grain weight under HNT conditions was demonstrated for field-grown rice when exposed to HNT stress from panicle initiation to maturity [18,25,26,27,58]. In agreement with this, we also found a negative HNT effect on the 1000-grain weight in both seasons under similar stress conditions as used in the previous studies.

Grain yield is influenced by carbon and nitrogen supply to the grain, which are affected by HNT [59]. Temperature-sensitive respiration, known to be increased under HNT (e.g., [28]), might have resulted in increased respiratory carbon loss, previously described to be important during the ripening period [60]. Dark respiration was also considered by other reports to be the main factor affecting biomass and yield under HNT conditions [12,20,25,61] and might be responsible for a decline in assimilation supply to developing grains [57].

This hypothesis is in agreement with the metabolite data obtained during the WS. We found a lower abundance of sucrose and the intermediates of glycolysis, such as glucose-6-phosphate, fructose-6-phosphate, glyceric acid-3-phosphate and glycerol-3-phosphate, whereas the monosaccharides glucose and fructose were increased in panicles. A similar decrease in sugar phosphates, but not in sucrose, was also reported for developing rice caryopses exposed to HNT during the milky stage [48]. Likewise, we also found a significant correlation between the magnitude of the changes in the fructose-6-phosphate content of the panicles under HNT conditions and the yield reduction in the WS. This emphasizes the importance of glycolysis for HNT tolerance in rice.

Glycolysis generates biosynthetic intermediates for respiration. Therefore, a high turnover of glycolysis, as indicated by reduced levels of intermediates, could be expected as respiration is highly increased under HNT. In addition, the products of glycolysis also feed into the TCA cycle, which was shown to be dysregulated in leaves under HNT conditions in climate chamber experiments [51,62]. On the other hand, no significant differences in the metabolites associated with the TCA cycle were found in the developing seeds of different rice cultivars under HNT [53]. Likewise, our data did not provide evidence for an altered TCA cycle under HNT conditions in either panicles or flag leaves.

Interestingly, the effects on glycolysis that we found in panicles in the WS were not observed in either flag leaves in our present study or previously in leaves of the vegetative stage [52]. Apparently, photosynthesis, which is unimpaired under HNT conditions, results in largely unaltered carbohydrate pools in leaves [28,52]. It is therefore reasonable to assume that the carbohydrate supply to the panicles is limiting for grain yield under HNT conditions. Lower sink capacity [26], possibly related to a reduction in the activity of enzymes involved in starch synthesis, has been discussed as a reason for the reduction in grain weight under HNT [63], which we have also observed. A further possibility is an impaired import of sucrose into the panicles under HNT conditions, as has been shown in rice under heat stress [44]. Further experiments will be necessary to test these hypotheses.

The larger reduction in grain yield in the WS compared to in the DS may nevertheless, at least in part, be related to carbohydrate availability. One factor may be faster development during a slightly shorter growing period in the WS, caused by higher daytime T_min_, preventing the accumulation of sufficient biomass, as shown previously in simulation models [54]. In addition, irradiance levels in the WS were much lower than in the DS, resulting in lower photosynthesis rates [64]. This may have led to a lower overall carbon supply for grain filling [20], leading to lower yield in the WS than the DS under control conditions and a more pronounced effect of HNT on yield in the WS [25] that was mitigated by the higher carbohydrate supply in the DS.

The amino acid alanine was among the significantly increased metabolites in panicles under HNT in the DS but not in the WS. Similarly, alanine was also increased under HNT during early seed development and in the early grain-filling stage in six rice cultivars [53] and in wheat spikes [65]. Alanine is synthesized by the enzyme AlaAT, which catalyzes the reversible synthesis of alanine and 2-oxoglutarate from pyruvate and glutamic acid [66]. It is therefore considered an intercellular nitrogen and carbon shuttle involved in both carbon fixation and nitrogen metabolism [67]. AlaAT is localized in various plant organs and is active in developing rice seeds [68]. The activity of AlaAT is increased in developing rice seeds under heat stress [48], and we observed a moderate increase under HNT conditions in the DS and a moderate decrease in the WS. While the overexpression of *AlaAT* from barley in rice or canola results in increased nitrogen uptake efficiency and a higher biomass and seed yield compared to in wild type plants [56,66,69,70,71], a rice mutant of *AlaAT1* exhibits decreased kernel weight [69]. The higher AlaAT activity in the DS may have led to increased nitrogen uptake and assimilation, as described for plants overexpressing *AlaAT* [56], while reduced activity in the WS may have had the opposite effect.

Another metabolite that was significantly increased in response to HNT in the DS, but not in the WS, specifically in panicles, was 3-cyano alanine. This compound is generated by the enzyme 3-cyano alanine synthase (EC 4.4.1.9) during the detoxification of cyanide, which is generated as a by-product of ethylene biosynthesis [72], when the precursor 1-aminocyclopropane-1-carboxylic acid (ACC) is converted into ethylene and hydrogen cyanide (HCN) by the activity of the enzyme ACC synthase [73]. The resulting 3-cyano alanine is then enzymatically converted to asparagine [74], which was also increased under HNT in the DS, indicating a functional detoxification process. Ethylene is a volatile plant hormone that is important for plant growth and development, and various biotic and abiotic stress responses [75]. HCN, on the other hand, is toxic to cells and therefore needs to be efficiently removed [74]. The lower amounts of 3-cyano alanine and asparagine in the panicles collected in the WS might point to a less efficient detoxification of HCN. This is in agreement with the finding that the magnitude of the reduction of both 3-cyano alanine and asparagine in panicles in the WS is significantly correlated with the reduction in grain yield in the WS observed across the eight cultivars. This may indicate that HCN toxicity plays an important role in the HNT sensitivity of panicles. Additionally, however, HCN may play a direct regulatory role in gene expression in low, non-toxic concentrations [76]. Whether this has any impact on HNT tolerance is currently not known.

Two polyols, arabitol and erythritol, were significantly increased in the flag leaves and panicles of almost all cultivars under HNT in both seasons. Both metabolites were also increased under HNT in the vegetative leaves of 12 rice cultivars, including the eight in the present study, in climate chamber experiments [51]. Polyols generally function as compatible solutes and antioxidants under abiotic and biotic stress conditions [77]. Furthermore, arabitol accumulates in flowering spikelets and developing seeds under combined drought and heat stress in the tolerant *aus* cultivar N22 and has a higher content in N22 compared to in sensitive cultivars in flag leaves in the field under control conditions [49]. Similarly, erythritol is accumulated in flowering spikelets and flag leaves under the same conditions, while it is decreased in developing seeds under combined drought and heat stress. Increased levels of erythritol were also found under drought conditions in Arabidopsis [78,79] and in flag leaves of 292 rice accessions [80]. In fact, arabitol and erythritol were both identified as potential metabolic markers for combined drought and heat tolerance [49], and erythritol content under control conditions was the best predictor of drought-induced yield loss in rice [80]. In the present study, however, no correlation between changes in arabitol or erythritol levels and grain yield under HNT was found. The accumulation of these sugar alcohols may therefore be an unspecific response to HNT stress.

## 4. Materials and Methods

### 4.1. Plant Material, Cultivation and HNT Stress Treatment

Eight *Oryza sativa* ssp. *indica* (IR123, IR62266-42-6-2, IR64 and IR72) and *japonica* (CT9993-5-10-1M, M202, Moroberekan and Taipei309) cultivars with different HNT tolerance in the vegetative stage under controlled environmental conditions [28] were used (Table 1). IR72, Taipei309 and Moroberekan were characterized as HNT tolerant; IR64, IR123 and CT9993-5-10-1M showed intermediate tolerance; and M202 and IR62266-42-6-2 were sensitive to HNT under these conditions [28]. The seeds for all cultivars were produced at the IRRI. The experiments were carried out during the WS and DS at the IRRI (14°11’N, 121°15’E, 21 MASL) in the Philippines. The seeds were pre-germinated in water after incubation at 50 °C for 3 d to break dormancy and were then sown in seeding trays. Fourteen-day old seedlings were transplanted to the field to a spacing of 0.2 × 0.2 m. The WS experiment was started in June 2011, with four seedlings per hill and each cultivar (42–48 hills) randomly assigned to two replicate plots per treatment. Phosphorus (15 kg·ha^−1^ p as single superphosphate), potassium (20 kg·ha^−1^ K as KCl), and zinc (2.5 kg·ha^−1^ Zn as zinc sulfate heptahydrate) were applied to all plots as a basal fertilizer a day before transplanting. Nitrogen (N as urea) was incorporated in four splits (30 kg·ha^−1^ as basal, 20 kg·ha^−1^ at mid-tillering, 30 kg·ha^−1^ at panicle initiation (PI), and 20 kg·ha^−1^ just before heading). For the DS experiment, seedlings were transplanted in a staggered approach with one batch in December 2013 and two batches in January 2014. The stagger sowing was based on the phenology data from the first experiment. Each cultivar was randomly assigned to five replicate plots per treatment with one seedling per hill and a total of 28–40 hills per plot. Basal fertilizer (30 kg·ha^−1^ P as single superphosphate, 40 kg·ha^−1^ K as KCl, and 5 kg·ha^−1^ Zn as zinc sulfate heptahydrate) was applied one day before transplanting. N fertilizer as urea was applied in four splits (45 kg·ha^−1^ as basal, 30 kg·ha^−1^ at mid-tillering, 45 kg·ha^−1^ at PI, and 30 kg·ha^−1^ just before heading).

During the day (6 a.m–6 p.m.), plants were exposed to ambient conditions (compare Figure A2 and Table 1). Overnight (6 p.m.–6 a.m.), plants were exposed to the temperature treatments in manually-covered tents with temperature-control devices as described previously [25]. Air conditioners were programmed to maintain the temperature setting at control (22 °C) or HNT (28 °C). Temperature and relative humidity were monitored by sensors connected to data loggers (HOBO, Onset Computer Corporation, Bourne, MA, USA). Temperature treatments started at the panicle initiation stage and lasted until physiological maturity (Figure A1). During the flowering stage, panicles that had flowered for at least 50% were identified and tagged. These were then collected, together with the corresponding flag leaves, the next morning just before the tents were opened (~4 a.m.–6 a.m.). All samples were collected in liquid nitrogen and stored at −80 °C until use.

### 4.2. Weather Data

Weather data (radiation, sunshine duration, rainfall, relative humidity, maximum temperature (T_max_) and minimum temperature (T_min_)) recorded by the IRRI wetland agrometeorological station were obtained from the IRRI Climate Unit.

### 4.3. Growth Analysis, Grain Yield and Yield Components

Twelve hills from each replicate plot were harvested at physiological maturity for the determination of plant height, tiller number, panicle number, and straw and rachis weight and processed for the analysis of yield components [81]. Sixty plants for the DS and 24 plants for the WS were considered for plant height, tiller number and panicle number. For the remaining parameters, two replicates pooled from twelve plants each were considered for the WS, and five replicates pooled from twelve plants each were considered for the DS. The number of panicles per hill was counted for the calculation of panicles per m^2^. Afterwards, plants were separated into straw and panicles and panicles were manually threshed. Filled and unfilled grains were submerged in water and separated with a seed blower. Filled, half-filled and empty grains were counted to obtain spikelets per m^2^, spikelets per panicle, seed set and 1000-grain weight. Total above ground biomass was determined from the dry weight of straw; rachis; and filled, half-filled and empty grains after drying at 70 °C until constant weight. The harvest index was calculated as the percentage of the dry weight of filled grains relative to the total above ground biomass. Plants from central areas of two m^2^ from each plot (two for the WS and five for the DS, per condition and cultivar) were also harvested for the determination of grain yield. Grain weight data were adjusted to a standard moisture content of 0.14 g H_2_O g^−1^.

### 4.4. Metabolite Profiling and Data Processing

A fraction enriched in small polar metabolites was prepared from 120 mg of fresh weight of snap-frozen and ground flag leaves or panicles from five biological replicates per cultivar and condition and analyzed by gas chromatography coupled to electron impact ionization-time of flight-mass spectrometry (GC/EI-TOF-MS) as described in [82]. Chromatograms were acquired and baseline corrected by the ChromaTOF software (LECO Instrumente GmbH, Mönchengladbach, Germany). TagFinder [83], the NIST08 software, (http://chemdata.nist.gov/dokuwiki/doku.php?id=start) (U.S. Department of Commerce, Gaithersburg, USA, MD) and the mass spectral and retention time index reference collection of the Golm Metabolome Database [84,85] were used for the manually supervised annotation of metabolites. Mass spectral intensities were normalized to fresh weight and ^13^C_6_-sorbitol (Sigma-Aldrich, Taufkirchen, Germany) as internal standard. The normalized data are available in Table S2.

Data pre-processing was done separately for both organs and included the omission of metabolites with more than 75% missing values and a missing value imputation for the remaining metabolites with half the minimum amount of the respective mass spectral intensity. Furthermore, contaminations were identified using hierarchical clustering and correlation matrices with a set of known contaminating compounds and removed. A batch effect correction of different measurements of the whole data set was performed using an ANOVA tool [86]. The intensities of each metabolite were divided by the median intensity across all measurements and log_2_-transformed to approximate a normal distribution. All presented metabolite data thus represent relative metabolite abundance measures. Outliers were detected with the function *grubbs.test* included in the R-package *outliers* [87] using a threshold of *p* < 0.0001. Finally, 132 metabolite intensities were detected for panicles and 161 metabolite intensities were detected for flag leaves for the DS, and 195 metabolites were detected for both tissues for the WS. For further analysis, the overlap of metabolites per tissue was determined, showing 69 metabolites for panicles and 76 metabolites for flag leaves.

To enable direct comparison, overlapping metabolites for each tissue between both experiments were determined, resulting in 69 metabolites for panicles and 76 for flag leaves.

### 4.5. Enzyme Activity

The activity of alanine aminotransferase (AlaAT, E.C.2.6.1.2) was measured according to a published method [88]. Ground panicle material (20 mg) was used from three biological replicates per cultivar and condition. In four cases (IR72, IR62266-42-6-2—C, HNT, Moroberekan—C, Moroberekan—HNT), only two replicates were available.

### 4.6. Statistical Analysis

PCA was perfomed with the R-package *pcaMethods* [89]. For the data processing and visualization, *R v3.4.2* [90] and *R-Studio v1.1.383* [91] were used including the following packages: *ggplot2* [92], *grid* [93], *gridExtra* [94] and *reshape2* [95].

Changes in metabolite content were investigated by calculating the log_2_ fold change between the averages of metabolite levels under control conditions in the DS compared to in the WS, or under HNT compared to under control conditions. Unpaired, two-sided t-tests were performed over all replicates, comparing control and HNT conditions to determine the statistical significance of the observed changes. For agronomic data, t-tests were applied for the DS. For the WS, only two replicates were available for most parameters and t-tests were only applied for plant height, tiller number and panicle number. To test the significance of the influence of genotype (G), treatment (T) and GxT interactions across all cultivars, a 2-way ANOVA design was used.

The statistical significance of differences in enzyme activity between control and HNT treatments were evaluated by an unpaired two-sided *t*-test, performed in RStudio [91].

Correlations between total grain yield reduction under HNT in the WS and the corresponding changes in metabolite content (log_2_ fold change) were done in R with the package *cor.test* using Spearman Rank Correlation with *p <* 0.05.

## Figures and Tables

**Table 1:** Experimental set-up for high night temperature (HNT) field experiments for eight contrasting *Oryza sativa* cultivars. Mean temperatures and relative humidity (RH) are given from the beginning of HNT treatment till the sampling time, when panicles reached 50% flowering.

	Experiment 1		Experiment 2	
Season	Wet		Dry	
Conditions	Control	HNT	Control	HNT
Cultivars	CT9993-5-10-1M			
	IR123			
	IR62266-42-6-2			
	IR64			
	IR72			
	M202			
	Moroberekan			
	Taipei309			
T_day_ (°C)	27.7		26.1	
T_night_ (°C)	22.2	27.6	22.2	27.8
RH (%)	98.3	89.4	96.4	80.9
Sampling time	Panicle at 50% flowering			
Samples	Flag leaves, panicles			

**Table 2:** Analysis of variance (ANOVA) on selected agronomic parameters. Sixty plants for the DS and 24 plants for the WS were considered for plant height, tiller number and panicle number. For the remaining parameters, two replicates pooled from twelve plants each were considered for the WS and five replicates pooled from twelve plants each were considered for the DS. Spikelets/panicle represents the number of spikelets per panicle. The seed set was calculated as follows: seed set (%) = filled grains/(filled+half-filled+unfilled grains) × 100. Harvest index was calculated as percentage of dry weight of filled grains relative to total above-ground biomass. The significance of the influence of genotype (G), HNT-treatment (T), season (S) or the interaction between two influences (GxT or TxS) on differences between HNT and control conditions across all eight cultivars is indicated by asterisks: 0.001 < ***; 0.001 > ** < 0.01; 0.01 > * < 0.05. ns—not significant. Original data for plant height, tiller number, panicle number and all yield components for the WS (2011) and the DS (2014) are available in Table S1.

Parameter	WS	WS	WS	DS	DS	DS	Both Seasons		
	G	T	GxT	G	T	GxT	T	S	TxS
Plant Height (cm)	***	ns	**	***	ns.	*	ns	ns	ns
Biomass g/m^2^	***	*	ns	***	ns	ns	ns	*	ns
Straw (g)	***	ns	*	***	ns	ns	ns	ns	ns
Rachis (g)	***	ns	ns	***	ns	ns	ns	*	ns
Tiller No	***	ns	ns	***	ns	ns	ns	**	ns
Panicle No	***	ns	ns	***	ns	ns	ns	**	ns
Panicle/m^2^	***	ns	ns	***	ns	ns	ns	**	ns
Spikelet/m^2^	***	*	ns	***	ns	ns	ns	***	ns
Spikelets/Panicle	***	**	ns	***	ns	ns	ns	***	ns
Seed set (%)	***	ns	ns	***	ns	ns	**	*	ns
Grain yield (g/m^2^)	***	***	ns	***	ns	ns	ns	**	ns
1000 grain weight (g)	***	***	***	***	***	ns	ns	***	ns
Harvest Index	***	***	ns	***	**	ns	ns	***	ns

## Supplementary Materials

The following are available online at https://www.mdpi.com/1422-0067/21/9/3187/s1.

## Abbreviations

ACC	1-aminocyclopropane-1-carboxylic acid
AlaAT	Alanine aminotransferase
DAS	Days after stress
DAT	Days after transplanting
DS	Dry season
DTR	Diurnal temperature range
FC	Fold change
G	Genotype
GABA	4-amino butanoic acid
GC-MS	Gas chromatography – Mass spectrometry
HCN	Hydrogen cyanide
HNT	High night temperature
IRRI	International Rice Research Institute
PC	Principal Component
PCA	Principal Component Analysis
RH	Relative humidity
S	Season
T	Treatment
TCA	Tricarboxylic acid
WS	Wet season
